# The Nightingale School and St. Thomas's Hospital

**Published:** 1917-11-10

**Authors:** 


### THE MATRONS' AND SISTERS' DEPARTMENT.

### THE NIGHTINGALE SCHOOL AND ST. THOMAS'S HOSPITAL.

THE intimate connection between St. Thomas's Hospital and Florence Nightingale lends the course of training provided for the nurses at this establishment an unique character. It will be remembered that the great foundress of modern nursing contemplated the existence of two distinct grades in her scheme for a national nursing service. The first grade would, she surmised, consist of "gentlewomen desirous to qualify themselves in the practice of hospital nursing with the object of filling superior situations in public hospitals and infirmaries, or of nursing the poor at their own homes under some organised system of district nursing." The second grade would be women qualifying with the aim of earning their living as private nurses or as subordinates to the first. When the national gift to Florence Nightingale rendered it possible for her to realise her aims in the Nightingale School for Nurses at St. Thomas's Hospital, it was natural that the education and training of the first grade of nurses should be her first care.

### THE FUNCTION OF THE NIGHTINGALE FUND.

The Nightingale nurses were trained in the first instance for public work. Time has wrought many changes. Almost every hospital in the kingdom now offers a course of training more complete than was the original counsel of perfection. Modern women, educated in elementary schools, are far better prepared for attendance at lectures than were many of the gentlewomen who became Nightingale probationers fifty years ago. The standard of instruction has risen all along the line, and the Nightingale School, if it was to fulfil the purposes of its foundress, needed to reconsider its curriculum and find its place in a new world. Was it right or reasonable that it should be content merely to subsidise the training of nurses on ordinary lines at a particular hospital? Was it not rather the duty of the authorities to look ahead to the future needs of the nursing service and aim at making the Nightingale Home a centre for preparing educated women to do their part in a national work?

### THE SISTER TUTOR.

It is indisputable that, with very few exceptions, the course of training offered in hospitals and infirmaries subordinates the instruction given, both theoretical and practical, to the exigencies of getting through the day's work. The arrangements made by the Committee of the Nightingale Fund with the authorities of St. Thomas's Hospital place the instruction given to the nurses on an equal footing with the services expected from them in the wards. They enter the service of the hospital as students destined to follow a definite course, and during their term of instruction, which lasts three years, their studies are under the immediate supervision of a sister tutor (holding nursing certificates), who herself lectures on certain subjects, is present at all lectures, gives classes on the subjects of the lectures, and helps the students in note-taking and the general mastery of their work. By appointing this highly qualified tutor, herself an experienced hospital sister, to take entire charge under the matron of all instruction given in the hospital, the authorities have ensured that the course, which is planned on lines of simplicity and thoroughness, shall be adapted to the needs of all the pupils, and that full advantage shall be taken of it. We are only too familiar with the high-sounding curriculum on paper prepared for the benefit of nurses who are manifestly incapable of mastering its intricacies. Only those who have themselves taught groups of probationers can realise the futility of much that is presented to them under the guise of training. Here, under the ægis of the Nightingale Home, we find a sincere attempt to organise on a practical basis the teaching subjects nurses need to know, and to allot sufficient time to serious study under a tutor who devotes her entire time and energies to directing her pupils.

*(To be continued.)*

